# Cesarean section and the gestational duration of subsequent pregnancies: A nationwide register-based cohort study

**DOI:** 10.1371/journal.pone.0317492

**Published:** 2025-02-05

**Authors:** Felix Evers, Christopher Flatley, Karin Ytterberg, Julius Juodakis, Pol Solé-Navais, Bo Jacobsson

**Affiliations:** 1 Department of Obstetrics and Gynecology, Institute of Clinical Sciences, Sahlgrenska Academy, University of Gothenburg, Gothenburg, Sweden; 2 Department of Obstetrics and Gynecology, Halland Hospital, Varberg, Sweden; 3 Department of Obstetrics and Gynecology, Sahlgrenska University Hospital, Gothenburg, Sweden; 4 Department of Genetics and Bioinformatics, Area of Health Data and Digitalization, Institute of Public Health, Oslo, Norway; Eulji University, REPUBLIC OF KOREA

## Abstract

**Introduction:**

Preterm delivery risk is increased for women with a previous cesarean section, which are becoming more common worldwide. However, this risk is based on studies which have not fully accounted for selection bias, and the studied outcomes have been limited to the study of early deliveries. This study aimed to determine the impact of previous delivery modes on the incidence of deviant duration of subsequent pregnancies.

**Material and methods:**

This retrospective cohort study used clinical data registered in the Swedish Medical Birth Register. 612 935 women with their first two pregnancies and 157 581 women with their first three pregnancies were included. The outcome was the gestational duration of the last pregnancy of the series, depending on previous delivery modes. The associations were analysed with multivariable logistic regression (with the gestational duration categorised) and survival models including Cox regression analyses.

**Results:**

When using standard logistic regression, previous cesarean section was associated with an increased risk of both spontaneous preterm birth (adjusted odds ratio [aOR] 1.67, 95% CI 1.57–1.77) and postterm birth (aOR 1.55, 95% CI 1.49–1.62). However, the more appropriate survival models only showed an association between cesarean section and longer gestational duration of the subsequent pregnancy (adjusted hazard ratio 0.72, 95% CI 0.71–0.73).

**Conclusions:**

The survival model handles bias related to management differences between the exposure and reference groups better than standard logistic regression, making it the statistical method of choice when conducting studies on this research topic. This study shows that the main association between cesarean section and the gestational duration of the subsequent pregnancy is the prolonging of it, which stands in contrast with previous research on the topic.

## Introduction

A pregnancy’s gestational duration is essential in determining the prognosis of a newborn [1, 2]. The World Health Organization (WHO) defines a pregnancy as full-term at 37+0 to 41+6 completed gestational weeks. Delivery outside this interval increases adverse outcomes, and preterm birth is the worldwide leading cause of perinatal mortality and lifelong neurological sequelae [3–7]. Even within the full-term interval, the outcome still depends on the exact gestational duration. Higher perinatal mortality is observed among newborns born outside the 39+0 to 40+6 interval [8–10].

A constantly increasing cesarean section (CS) rate is reported worldwide [11], which can be explained by a number of obstetrical and sociological factors. In Sweden, the CS rate was 17.9% during 2008–2017 [12]. Compared to vaginal delivery, CS is associated with increased incidence of several adverse maternal and neonatal outcomes [13, 14]. While some CSs are necessary, this creates an incentive to limit birth through CS when the indication is relative.

A meta-analysis found an adjusted relative risk of 1.12 (95% CI 1.01–1.24) for preterm birth after CS [15]. Reports have since shown an association between late-stage CS and preterm birth [16–20]. There has been a discussion regarding the association between CS and preterm birth, partly because they share risk factors but also due to the inclusion of both spontaneous and iatrogenic onsets of births in the studied cohorts [21]. Little focus has been directed towards the links between the previous delivery mode and the duration of subsequent pregnancies, missing the opportunity to study other time-points of pregnancy that are also crucial for maternal and fetal health.

The type of onset of birth is affected by previous obstetrical history. Women with a previous CS are more likely to have a repeat CS [21]. As an iatrogenic onset of birth, such as a planned CS, has a direct impact on the gestational duration of the pregnancy, the type of onset of birth has to be adequately accounted for when studying relationships between previous delivery modes and gestational duration of subsequent pregnancies.

This study aims to determine the impact of previous delivery modes on the incidence of deviant gestational duration of subsequent pregnancies. To do this we have compared women with previous CS(‘s) versus women with previous vaginal delivery(ies), in mothers who had two or more pregnancies. We give specific attention to the order of the previous delivery modes when analysing a woman’s first three pregnancies, covering a larger part of the woman’s child-bearing years. To account for the type of onset of birth, we use survival models as a new approach to evaluate the net effect of the previous delivery modes on the gestational duration of the subsequent pregnancies.

## Materials and methods

This is a register-based cohort study using pregnancies in the Swedish Medical Birth Register (MBR) [22]. The Swedish National Board of Health and Welfare maintains the register and covers 98–99% of all deliveries nationwide. It provides information about the mother, obstetric history, current pregnancy, delivery, and data concerning the baby. The data are recorded by hospital staff [23]. Individual-level data were pseudonymized for research use and provided under a permit from the Swedish National Board of Health and Welfare.

The exposure in this study was the previous delivery mode: CS, vaginal delivery, or a combination of the two. The record of a CS is generated as the surgery is documented in the patient’s medical record. The MBR does not provide information about the stage of labor when the CS is performed, or reliable information about whether the CS was planned or acute, therefore both planned and acute CSs are included in this study. The onset of birth is classified as either spontaneous or iatrogenic (planned CS or induction of labor).

**Fig 1:**
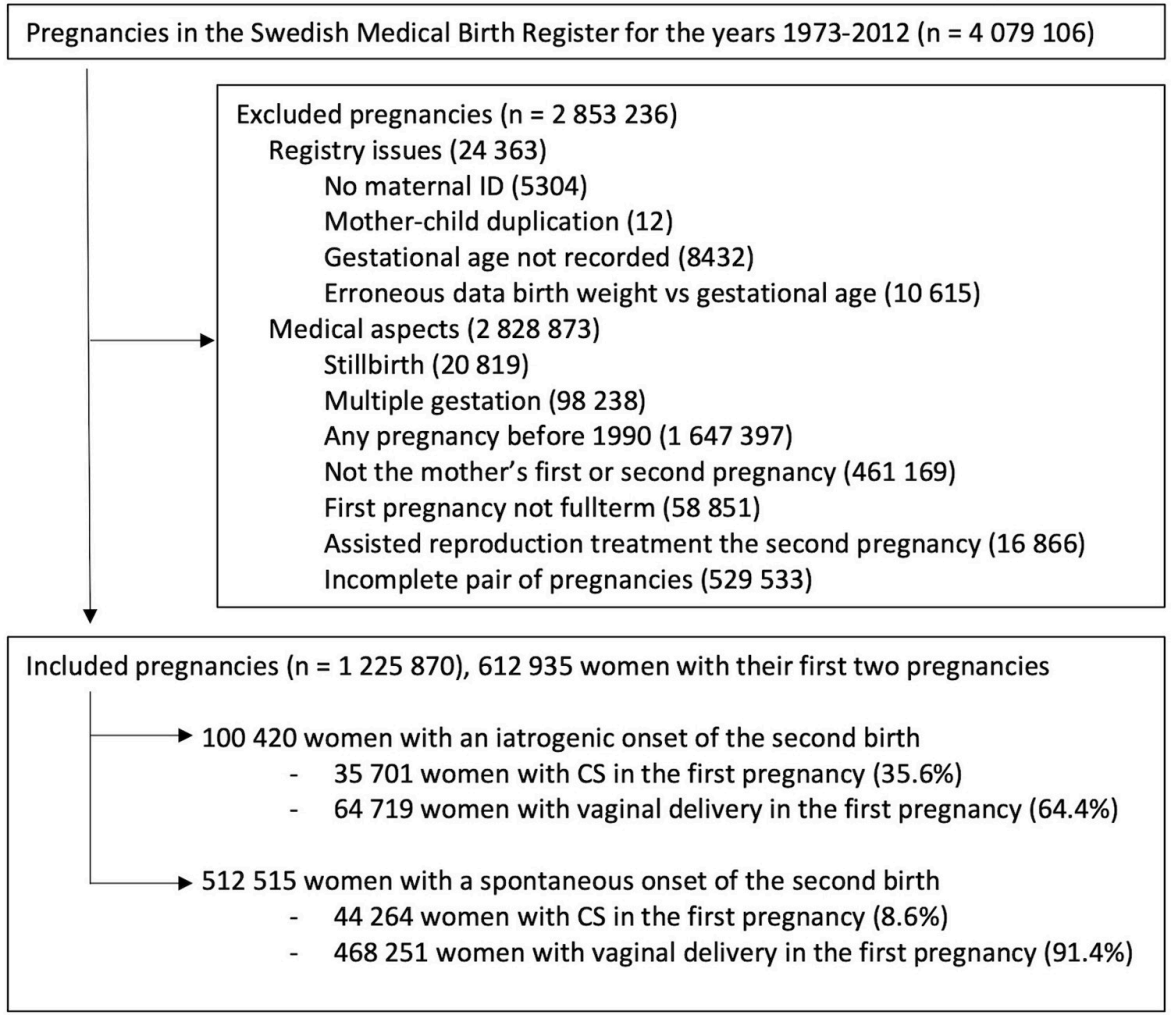
Study population, first two pregnancies. Inclusion and exclusion flowchart. *CS*: cesarean section.

**Fig 2:**
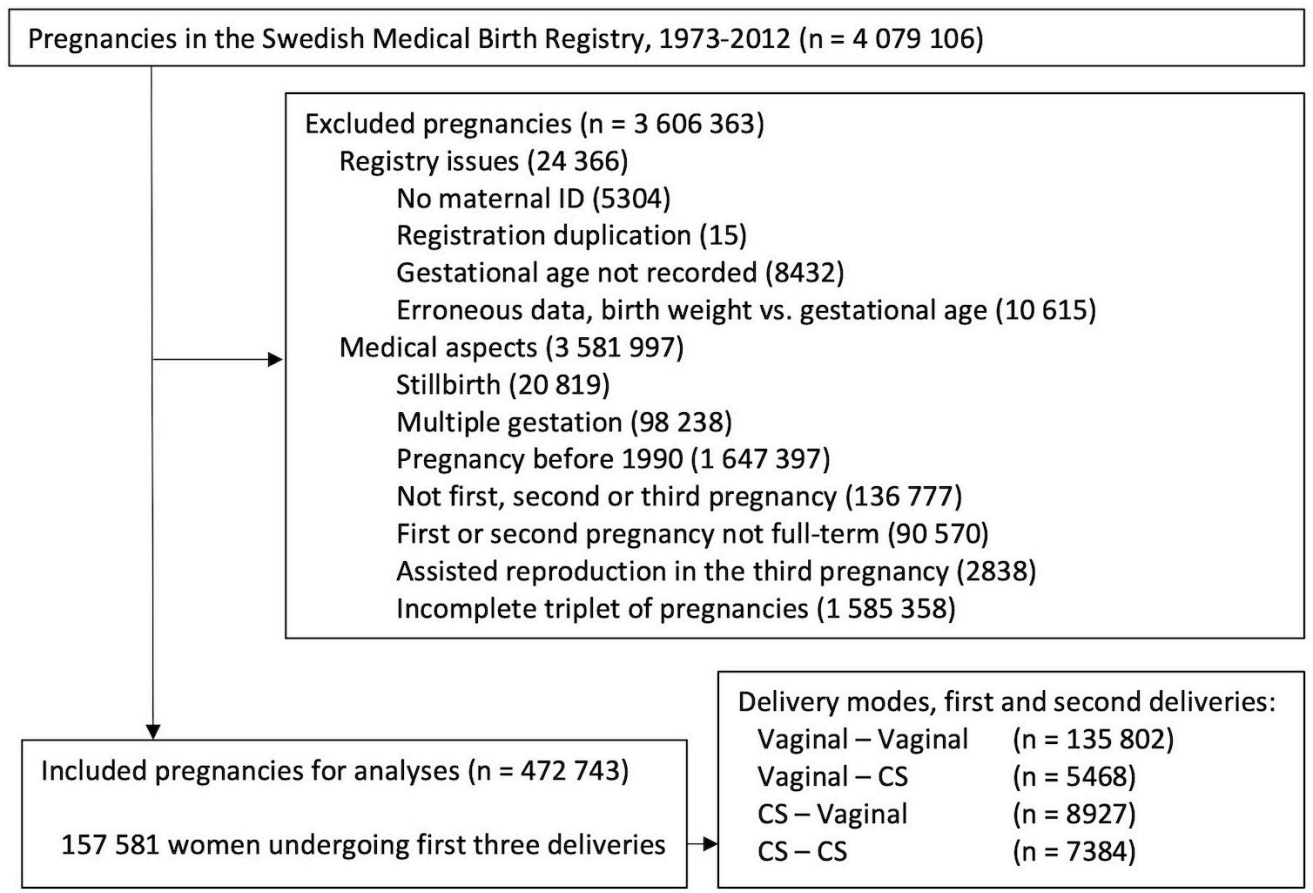
Study population, first three pregnancies. Inclusion and exclusion flowchart. *CS*: cesarean section.

The included cohorts were mothers with their first two pregnancies recorded in the MBR, and another analogous, largely overlapping cohort with mothers with their first three pregnancies recorded. We had access to data between 1973–2012. The type of onset of birth was not recorded before 1990, excluding earlier pregnancies. Registry issues leading to exclusion were absent or duplicated maternal ID, missing information of the gestational duration of the pregnancy, or unreliable registration of the gestational duration based on a comparison with the child’s birth weight. Further exclusions were made for stillbirth, multiple gestation, previous preterm birth and use of assisted reproduction techniques in the outcome pregnancy. The inclusion and exclusion criteria are presented in Figs 1 and 2.

The outcome was the gestational duration of the last pregnancy of the series. In the MBR, the best estimate of the gestational duration is based on ultrasound or the last menstrual period, according to a hierarchical set of rules [23]. The majority of the pregnancies included in this study (86.9–88.4%) had their gestational duration estimated by ultrasound. The gestational duration was stratified based on the WHO classification of preterm birth: preterm <37+0, late preterm 34+0–36+6, moderate preterm 32+0–33+6, very preterm 28+0–31+6 and extremely preterm <28+0 gestational weeks [3]. Additional strata were created for gestational weeks 37+0–37+6, 38+0–38+6, 41+0–41+6 and >41+6 [8–10]. This allowed analyses of the association between previous delivery modes and delivery within a specific and clinically relevant gestational interval, compared to the reference interval of 39+0–40+6 weeks used in this study, and to explore the possibility of non-linear effects.

Multivariable logistic regression analyses were conducted with different gestational duration intervals as outcome when analysing pairs of pregnancies, and preterm birth as outcome when analysing trios of pregnancies. For the regression analyses, only series of pregnancies where the last pregnancy had a spontaneous onset of birth were included. Statistical significance was set at *p* < 0.05, with a Bonferroni-corrected threshold of 0.0056 (0.05/9) for multiple testing of different gestational duration intervals. The regression models were adjusted for: maternal age, body mass index in early pregnancy, mother born outside Sweden, smoking, diabetes (including gestational diabetes), hypertensive disorder (including preeclampsia), baby’s sex, congenital malformation and small or large for gestational age. For the extremely preterm group, diabetes, hypertensive disorders and large for gestational age were not included due to too few cases. Small and large for gestational age were defined as birth weight deviating by more than two standard deviations from the mean for the gestational age according to Marsal, the classification system used in clinical practice in Sweden [24]. Missing body mass index data (12% of records) was handled through mean imputation. The ICD codes used to define the diagnosis-based variables are provided in Supporting Information S1 Table. Statistical analyses were performed with R version 4.1.2. The code is available on https://github.com/PerinatalLab/prevCS-PTD.

The Swedish obstetrical policy is to recommend a planned CS in the third pregnancy after two previous CSs, while recommending a vaginal birth after one previous CS. This clinical practice has consequences when studying this research topic. Excluding women with an iatrogenic onset of birth in the outcome pregnancy would lead to an overrepresentation of women with delivery in early gestational weeks, since they are the ones who had a spontaneous onset of birth before their scheduled CS date. This inflates the odds ratios for preterm delivery after one previous CS, and in particular after multiple previous CSs. To reduce this bias, survival analyses were performed (Kaplan-Meier curves calculated and Cox regression analyses to adjust for the same covariates as in the logistic regression models) where mothers with an iatrogenic onset of the last delivery of the series were included, but censored at the time of birth. This allowed including these mothers, despite lacking knowledge about how long the gestational duration of those pregnancies would have been had there not been an obstetrical intervention ending the pregnancy. The censoring time marks the shortest possible gestational duration of the pregnancy if it would have had a spontaneous onset of birth. This design extracts the most possible information out of each pregnancy while allowing us to keep the original group sizes intact, thereby handling the problem of inflated odds ratios of spontaneous preterm birth for women with a previous CS.

Ethical approval for this study was granted by the Regional Ethical Review Board in Gothenburg on July 29, 2013 (Dnr 576–13) and by the Swedish Ethical Review Authority on October 12, 2022 (Dnr 2022-05062-02). Participant consent was waived for this study.

## Results

### Descriptive characteristics of the cohorts

**Fig 3:**
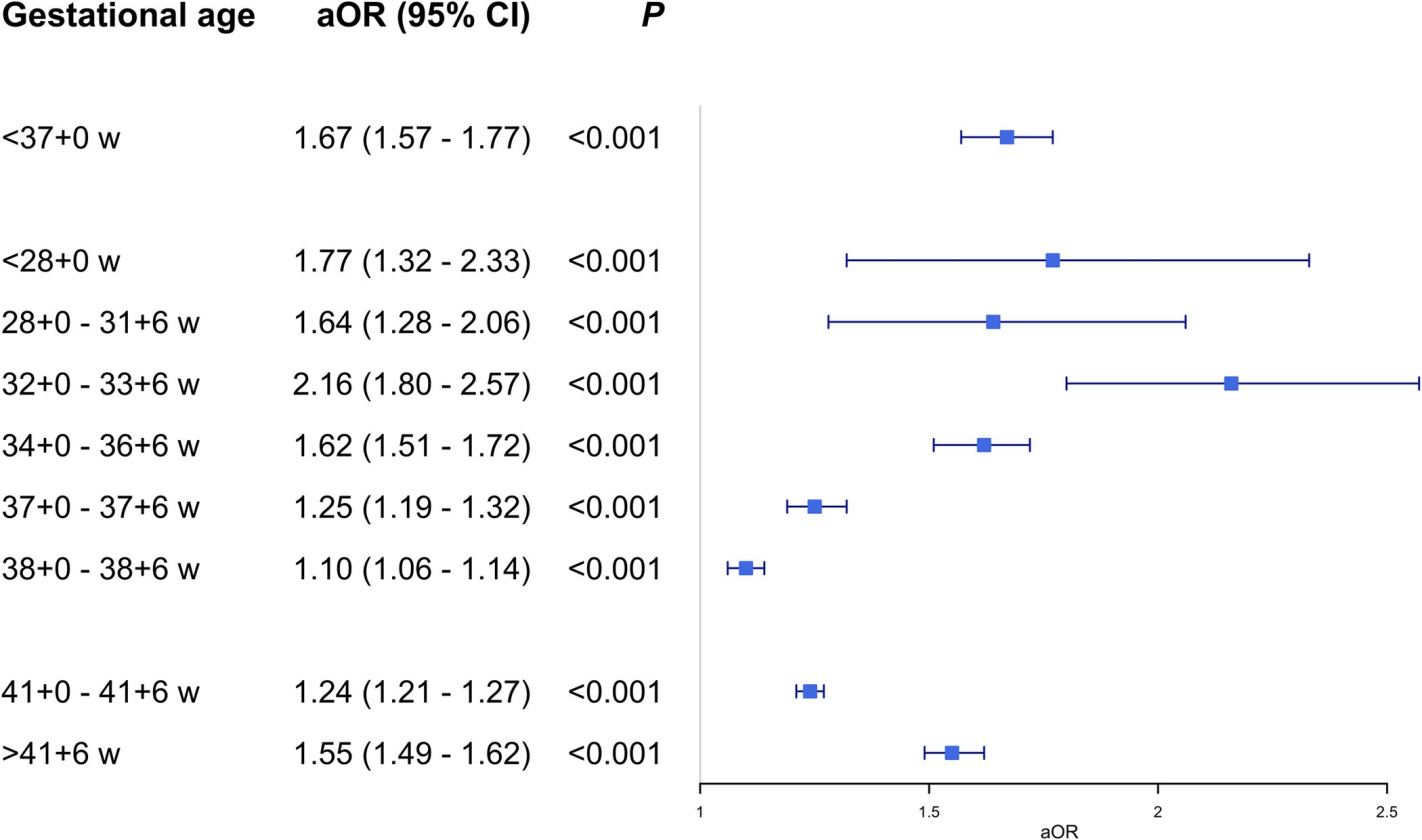
Multivariable logistic regression analyses, first two pregnancies. Adjusted odds ratio for gestational age at birth within the specified interval, in the second pregnancy after cesarean section in the first pregnancy. Only mothers with spontaneous onset of birth in the second pregnancy were included. *aOR*: adjusted odds ratio.

The cohort with two pregnancies consisted of 612 935 women (full cohort), of which 512 515 had a spontaneous onset of the second birth (subcohort) (Table 1). The first delivery was by CS in 79 965 pregnancies (13.0%) in the full cohort and in 44 264 pregnancies (8.6%) in the subcohort of only spontaneous onsets of the second birth. The distribution of the outcome variable, the gestational duration of the subsequent pregnancy categorised, is presented in the lowermost part of Table 1. Analysing the subcohort, the proportions of deliveries within the different gestational age intervals differ between the groups: the previous-CS group has higher birth rates in earlier and later weeks (Fig 3). Other differences between the groups are higher prevalence of diabetes, hypertensive disorders, fetal malformations, growth alterations and baby boys in the previous-CS group. Moreover, the birth rate through CS in the second pregnancy after a CS is 26.4% compared to 1.9% after a vaginal delivery.

**Table 1:** Cohort characteristics, first two pregnancies.

Cohort of mothers with two consecutive pregnancies	Full cohort, any onset of the second birth (n = 612 935)		Subcohort, only spontaneous onsets of the second birth (n = 512 515)	
Delivery mode 1^st^ pregnancy	Cesarean section	Vaginal	Cesarean section	Vaginal
n (%)	79 965 (13.0)	532 970 (87.0)	44 264 (8.6)	468 251 (91.4)
**Pregnancy 1**				
Maternal age, years (mean, SD)	28.2 (4.6)	26.6 (4.4)	27.9 (4.5)	26.6 (4.4)
Gestational duration, days (mean, SD)	280.9 (10.8)	281.3 (8.8)	280.4 (10.6)	281.0 (8.7)
Body mass index, kg/m^2 (mean, SD)	24.4 (4.0)	23.5 (3.3)	24.1 (3.7)	23.4 (3.3)
Mother not born in Sweden (%)	15.4	14.7	14.8	14.8
Smoking (%)	9.9	11.2	7.3	8.6
Diabetes (%)	1.9	0.6	1.2	0.5
Hypertensive disorder (%)	7.5	4.0	6.9	3.6
Sex of child, boy (%)	54.3	51.0	54.2	51.0
Fetal malformation (%)	5.6	3.3	6.1	3.3
Small for gestational age (%)	4.5	2.3	4.6	2.3
Large for gestational age (%)	4.3	1.4	3.1	1.2
**Pregnancy 2**				
Maternal age, years (mean, SD)	31.3 (4.6)	29.7 (4.5)	30.9 (4.5)	29.7 (4.5)
Gestational duration, days (mean, SD)	277.6 (11.7)	279.8 (10.5)	280.5 (10.8)	280.2 (9.5)
Ultrasound-based gestational duration (%)	87.6	86.8	87.6	86.8
Interpregnancy interval, months (mean, SD)	28.6 (22.7)	28.5 (23.4)	27.1 (21.0)	27.8 (22.5)
Body mass index, kg/m^2 (mean, SD)	25.3 (4.6)	24.2 (4.0)	24.9 (4.3)	24.0 (3.9)
Smoking (%)	7.5	8.7	7.3	8.6
Diabetes (%)	2.6	0.9	1.7	0.7
Hypertensive disorder (%)	3.5	2.1	2.1	1.2
Sex of child, boy (%)	51.3	51.4	50.2	51.1
Fetal malformation (%)	3.5	3.1	3.2	3.0
Small for gestational age (%)	1.6	1.2	1.3	0.8
Large for gestational age (%)	7.0	4.2	4.4	3.8
Cesarean section delivery (%)	53.4	5.7	26.4	1.9
Spontaneous onset of birth (%)	55.4	87.8	100	100
Preterm birth, <37+0 (%)	3.5	2.7	3.2	2.0
<28+0 (%)	0.1	0.1	0.1	0.08
28+0–31+6 (%)	0.3	0.2	0.2	0.1
32+0–33+6 (%)	0.4	0.3	0.3	0.2
34+0–36+6 (%)	2.7	2.1	2.5	1.7
37+0–37+6 (%)	4.8	3.6	3.6	3.2
38+0–38+6 (%)	24.7	12.0	10.2	10.5
39+0–40+6 (%)	44.4	57.4	54.5	60.6
41+0–41+6 (%)	15.7	18.5	22.5	19.6
>41+6 (%)	6.9	5.7	6.1	4.2

The two columns to the right represent the subcohort consisting only of mothers who had a spontaneous onset of the second birth. Since this subcohort has quite different characteristics compared to the group as a whole (the two columns to the left), it underscores the importance in taking the onset of birth into consideration when designing studies on this subject. *SD*: standard deviation.

The cohort with three pregnancies consisted of 157 581 women (Table 2). The onset of the third birth has a big impact on the observed proportion of preterm births: for women with two previous CSs, the rate of preterm birth is 5.1% for the group as a whole, but 18.1% if only women with a spontaneous onset of the third birth are included. The Swedish obstetrical practice of recommending vaginal delivery after one previous CS is illustrated by the birth rates through CS being 26.4% for the second delivery after one CS, and 98.7% for the third delivery after two CSs.

**Table 2:** Cohort characteristics, first three pregnancies.

Cohort of mothers with three consecutive pregnancies (n = 157 581)				
Previous delivery modes	Vaginal-Vaginal	Vaginal-CS	CS-Vaginal	CS-CS
n (%)	135 802 (86.2)	5468 (3.5)	8927 (5.7)	7384 (4.7)
**Pregnancy 1**				
Gestational duration, days (mean, SD)	281.2 (8.7)	282.6 (9.1)	280.2 (10.7)	282.1 (10.9)
**Pregnancy 2**				
Gestational duration, days (mean, SD)	281.0 (8.1)	275.6 (9.5)	282.2 (8.3)	276.3 (9.6)
**Pregnancy 3**				
Gestational duration, days (mean, SD)	280.3 (10.6)	274.9 (11.9)	279.3 (11.6)	268.9 (8.2)
Gestational duration based on ultrasound (%)	87.8	87.9	87.9	86.9
CS delivery (%)	5.6	57.6	16.7	98.7
Spontaneous onset of delivery (%)	84.4	41.7	76.0	8.2
Preterm birth, <37+0 (%)	2.6	4.1	3.2	5.1
**Pregnancy 3, spontaneous onset**				
Gestational duration, days (mean, SD)	280.8 (9.3)	279.2 (12.6)	280.6 (10.0)	265.7 (16.3)
Gestational duration based on ultrasound (%)	87.8	88.4	88.2	88.6
CS delivery (%)	2.0	17.8	5.9	86.1
Preterm birth, <37+0 (%)	1.8	4.2	2.0	18.1

Characteristics of the three-pregnancy cohort depending on delivery modes the first two pregnancies. *CS*: cesarean section. *SD*: standard deviation.

**Fig 4:**
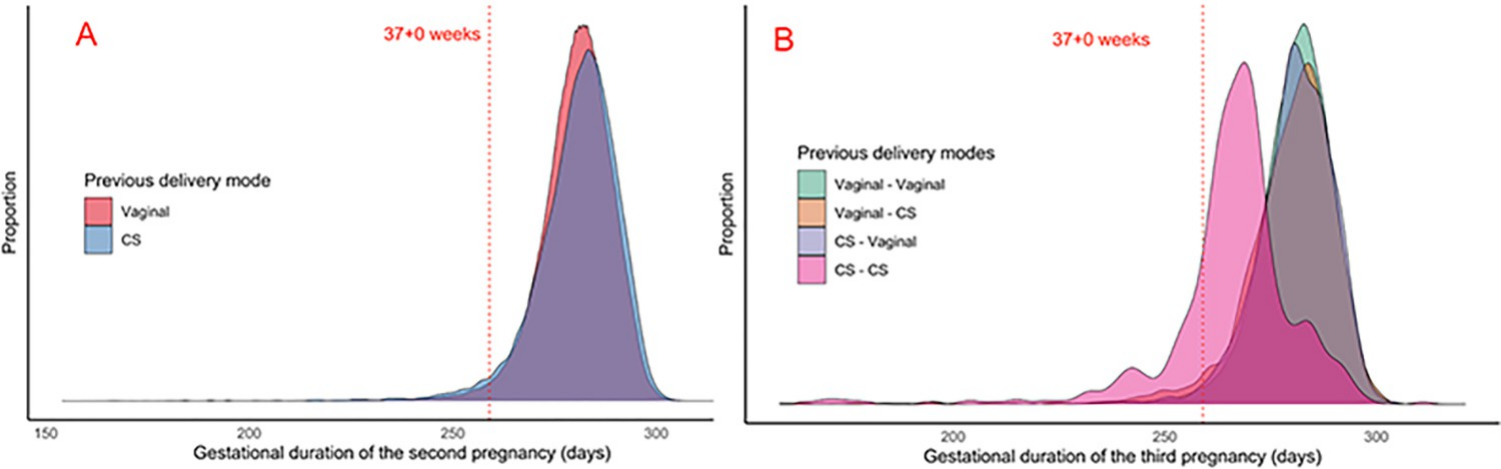
Gestational duration of the second and third pregnancies. The distributions of gestational duration of the second and third pregnancies, stratified by previous delivery modes. Only mothers with spontaneous onset of birth in the final pregnancy of the series. *CS*: cesarean section.

For two consecutive pregnancies, a previous CS shifted more weight to the extremes of the distribution of the gestational duration of the second pregnancy (Fig 4(A)). For three consecutive pregnancies (Fig 4(B)), the seemingly shorter gestational duration of the third pregnancy for the group with two previous CSs stands out. However, the graphs only include women with a spontaneous onset of the last birth and illustrate the bias discussed before (overinclusion of women with delivery in early gestational weeks), which happens when excluding mothers with an iatrogenic onset of the outcome pregnancy. This illustrates the difficulty in separating the true impacts of previous CSs on the duration of future pregnancies from technical artifacts.

### Logistic regression shows an increased risk of spontaneous preterm and postterm birth after previous cesarean section

**Fig 5:**
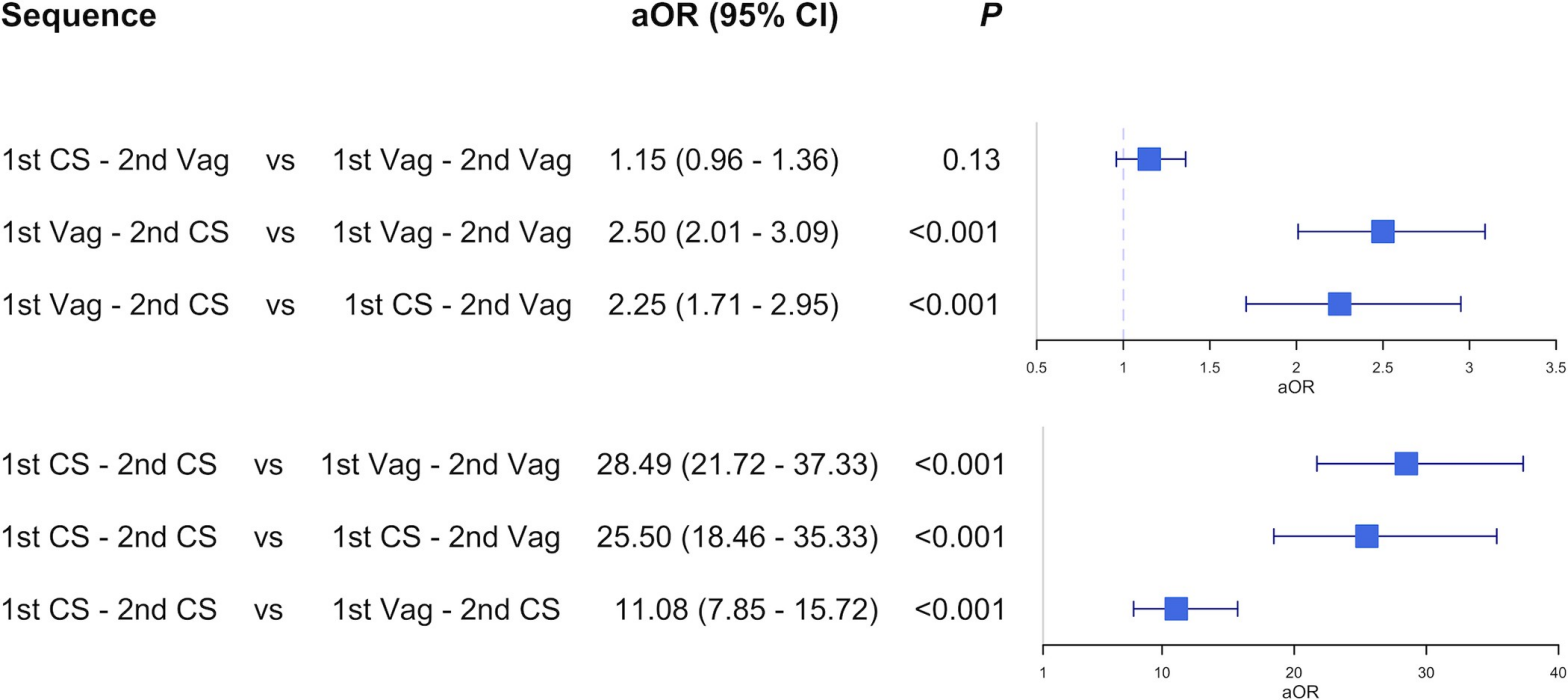
Multivariable logistic regression analyses, first three pregnancies. Adjusted odds ratio for preterm birth in the third pregnancy for the specified comparison between different sequences of previous delivery modes. Only mothers with spontaneous onset of birth in the third pregnancy. *CS*: cesarean section, *Vag*: vaginal delivery.

For the cohort with two consecutive pregnancies (Fig 3), previous CS was associated with a higher risk of spontaneous preterm and postterm birth, with larger effect sizes for intervals further away from term (logistic regression, all *p*-values highly significant, <0.001). The three-pregnancy cohort (Fig 5) also shows an association between CS and subsequent spontaneous preterm birth. The risk of spontaneous preterm birth is however not higher in the third pregnancy if a mother had a CS in the first pregnancy followed by a vaginal birth in the second pregnancy, compared to mothers with two previous vaginal deliveries (aOR 1.15, 95% CI 0.96–1.36). This specific result stands out in relation to the rest of this study, where a CS consistently has an association with spontaneous preterm birth in a subsequent pregnancy.

As a sensitivity analysis, regression models were created which only included pregnancies where the gestational duration was estimated by ultrasound. The results (Supporting Information S1 and S2 Figs) show no meaningful differences compared to the models relying on the best estimate of the gestational duration.

### Survival analyses show the link between cesarean section and prolonged pregnancy

The exclusion of women with an iatrogenic onset of birth of the outcome pregnancy in a logistic regression model leads to falsely high odds ratios for delivery before a scheduled CS date. This problem is clearly seen with the unreasonably high odds ratios in Fig 5 (birth distribution if Fig 4(B)). The results in Fig 3 have the same problem (birth distribution in Fig 4(A)). Our solution to the bias generated by the unfortunate combination of the clinical management policy together with logistic regression is to use survival models.

**Fig 6:**
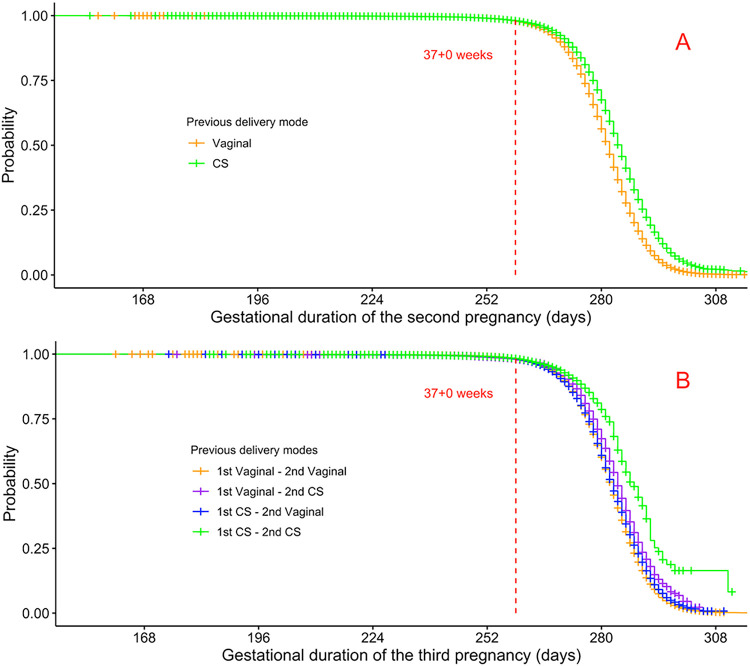
Kaplan-Meier curves, series of two (A) and three (B) pregnancies. The curves illustrate the estimated probabilities of “survival” (i.e. the curve value at *t* days is the probability that a pregnancy lasts longer than *t*) depending on previous delivery mode. All mothers regardless of the onset of the final birth of the series are included, the mothers with an iatrogenic onset of the final birth are censored from the gestational age at birth.

The Kaplan-Meier curves of Fig 6(A) show a prolonged pregnancy for the previous-CS group compared to the previous-vaginal delivery group. In Fig 6(B), the analogous survival analysis for the cohort with three consecutive pregnancies shows the scenario of two previous CSs giving the longest prolongation of the third pregnancy, while the scenario of two previous vaginal deliveries corresponds to the shortest gestational duration of the third pregnancy. The scenario of a CS in the first delivery followed by vaginal delivery in the second pregnancy yet again shows a very slight difference versus two previous vaginal deliveries, consistent with the results of the logistic regression model (Fig 5) that showed no statistically significant association with spontaneous preterm birth for that group.

The Cox regression models (Table 3) calculate the adjusted hazard ratios (aHR) between the different scenarios of previous delivery modes using the same covariates as the previous logistic regression models. For two pregnancies (Fig 6(A), Table 3), the aHR for previous CS versus previous vaginal delivery is 0.72 (95% CI 0.71–0.73), i.e., a net effect of a prolongation of the pregnancy following a CS. For three pregnancies (Fig 6(B), Table 3), the largest prolongation of the third pregnancy is seen when comparing two previous CSs versus two previous vaginal deliveries (aHR 0.64, 95% CI 0.59–0.69). A very slight prolongation is seen when comparing the scenario of a CS followed by a vaginal delivery versus two previous vaginal deliveries (aHR 0.95, 95% CI 0.93–0.98).

**Table 3:** Cox regression models.

Previous delivery modes	Reference group	aHR	95% CI	*p*-value
**Two consecutive pregnancies**				
CS	Vaginal	0.72	0.71–0.73	<0.001
**Three consecutive pregnancies**				
1^st^ Vaginal– 2^nd^ CS	Vaginal—Vaginal	0.80	0.77–0.84	<0.001
1^st^ CS– 2^nd^ Vaginal	Vaginal—Vaginal	0.95	0.93–0.98	<0.001
1^st^ CS– 2^nd^ CS	Vaginal—Vaginal	0.64	0.59–0.69	<0.001

The Cox regression models are adjusted for the same covariates as the previous logistic regression models. The hazard ratio reflects the likelihood of delivery at a certain time versus the reference group. *CS*: cesarean section; *CI*: confidence interval; *aHR*: adjusted hazard ratio.

## Discussion

### Main findings

Using survival models, the main result of this study is that there is an association between a previous CS and a prolongation of the subsequent pregnancy. While logistic regression shows an increased risk for both spontaneous preterm birth (aOR 1.67, 95% CI 1.57–1.77) and spontaneous postterm birth (aOR 1.55, 95% CI 1.49–1.62) in the second pregnancy after a CS in the first pregnancy, this is not the preferred way of analysing this research question since it overestimates the risk of preterm birth in the second pregnancy. Further, a CS in the first pregnancy followed by a vaginal birth in the second pregnancy is not associated with an increased risk for spontaneous preterm birth in the third pregnancy, compared to two previous vaginal deliveries (aOR 1.15, 95% CI 0.96–1.36). Previous studies have described the association between CS and subsequent preterm birth, but the main novel finding of this study is the association between CS and subsequent prolonged pregnancy, with an extensive methodological elaboration on the importance of taking the type of onset of birth in consideration when studying this subject.

### Strengths and limitations

A strength of this study is the reliability and the level of detail of the included data [22, 23]. A benefit of performing this study on Swedish patient data is the relatively high rate of vaginal birth after CS in Sweden (73.6%) compared to other countries [25]. Even if we have used a survival model to adjust for the bias due to a larger proportion of iatrogenic onsets of birth in the exposure group, the robustness of the results is helped by the very high rate of vaginal birth after one CS in Sweden, since it reduces the uncertainty of the results due to a lesser need for the model and its impacts on the observed data. The study covers a large time period and is nationwide, yet the clinical practice was similar over the entire country during the whole time frame of the study. The benefit of performing this study before the CS rate has increased even further that today is that it keeps problems generated by management differences between the exposure and reference groups as small as possible. A reasonable assumption is that Sweden will follow the global trend with an increased CS rate, and therefore it is likely that the use of advanced statistical maneuvers will become even more important, and unfortunately create even more interpretation problems than seen today.

There is, despite our best efforts to minimize it, still a risk of residual confounding that might have affected the observed results. The most significant risk we have identified is the possibility of confounding by indication, because even if we have tried to identify a reasonably homogenous group of women for inclusion and then adjust statistically for covariates differing between the groups, there will on the individual level always be a reason why the specific delivery mode was chosen, generating a difference between the exposure and reference groups that is unlikely to be fully resolved in any observational study design. A large portion of the potential bias that could be related to this phenomenon is, in our opinion, avoided through the inclusion of relatively healthy previous pregnancies, with the most critical step being the decision to only include previous-term pregnancies. Another limitation is the possibility that the outcome of previous pregnancies may affect how likely the woman is to have another pregnancy, which would alter the results of studies such as this, a limitation that cannot be resolved within a birth cohort. Other limitations are the lack of information in the MBR about the indication for the CS and at which stage of labor it was performed.

### Interpretation

The worldwide CS rate is expected to keep increasing, leading inevitably to an increase in the negative consequences of CSs. The aim of this study was to investigate the link between previous delivery modes and the incidence of deviant duration of subsequent pregnancies. While at first glance, our results seem coherent with previous studies, the survival analysis of this study changes that interpretation. Including only mothers with a spontaneous onset of the subsequent birth seems reasonable since we are mainly interested in any biological effects of a previous CS on its gestational duration. However, such selection generates a bias because the group of women with a previous CS are more likely to have an iatrogenic onset of the next pregnancy (45% vs 12%, Table 1), and therefore, spontaneous births will per definition occur before the date of an iatrogenic onset. Excluding women with an iatrogenic onset of birth, while keeping those with a spontaneous onset of birth, will exclude a relatively larger fraction of women from the previous-CS group, making that group seem smaller than it really is, and with a heavy burden of preterm birth, overestimating the risk of preterm birth for women with a previous CS. The apparent effect of a previous CS leading to increased preterm birth risk will largely be due to the study design, as the increased risk of spontaneous preterm birth after previous CS is no longer observed when applying survival models. In these, mothers with an iatrogenic onset of birth in the outcome pregnancy are kept; in effect, they are “followed-up” until the time of their CS. While we do not know their “natural” delivery date, we know that most of these mothers delivered around term, i.e. they had a low risk of spontaneous preterm birth. By including these mothers, the survival analysis shows that previous CS does not increase the risk of preterm birth. Thus, retaining the full group, i.e. not excluding the mothers with an iatrogenic onset of the next delivery, is essential. This result implies that the increased risk observed with logistic regression in this and other studies is likely attributable to a technical artefact rather than a true consequence of previous CS.

In fact, with appropriately chosen analysis models, we instead see an association in the other direction–between a previous CS and a prolonged subsequent pregnancy. To our knowledge, this association has not been reported before, as previous research has focused on the increased risk of preterm birth subsequent to CS [26, 27]. A possible explanation to this phenomenon could be that the physiological alteration of uterine function resulting from a CS may exacerbate uterine intolerance or, in the case of prolonged pregnancies, diminish the sensitivity of uterine load [28]. One speculation is that the CS might cause a stiffness in the uterus during the healing process, with an ensuing problem in failing to recognize the signals of onset of labor. This might be due to either the scar itself having poor contractile properties, or the failure of the area to allow effective signaling between the body and the cervix of the uterus, altering the maturation of the cervix and the onset of birth mechanism.

Another interesting finding seen in the cohort with three pregnancies was that a vaginal delivery in the second pregnancy after a CS in the first pregnancy proved to be no different in terms of association with subsequent spontaneous preterm birth in the third pregnancy compared to having had two previous vaginal deliveries. It seems that a vaginal birth after a CS could possibly be considered “a stress test” of uterine function in terms of achieving vaginal delivery, selecting a subgroup of women with lower risk of developing complications due to their previous CS, perhaps due to a smaller or more fortunately placed incision.

Regarding differences between the previous CS- and previous vaginal delivery groups, the overrepresentation of baby boys in the CS group is noteworthy. This discrepancy might possibly be explained by the fact that baby boys seem to be at generally higher risk of fetal distress [29, 30]. The higher prevalence of SGA, LGA, preeclampsia, and diabetes in the CS group are expected, as these factors are known to increase the likelihood of developing fetal distress [26, 31, 32]. Having taken all this into account, the differences between the cohorts should be considered to be minor and adjusted for.

Since previous studies have seen increased risk for subsequent spontaneous preterm births after late-stage CSs [16–20], a subanalysis on late-stage CSs in our cohort might have added valuable information, particularly if late-stage CS also increases the risk of subsequent delayed onset of birth. For future studies, investigating if there is any evidence of alterations in uterine function after previous surgery unrelated to the scar itself, would be of interest. That could possibly be grounds for re-evaluating the results of this study.

## Conclusion

The issue if there is a relationship between delivery through CS and an increased risk of subsequent preterm delivery is important, since that would be a severe consequence of a medical intervention that is potentially avoidable. Through a new methodological approach, we suggest survival models as the preferred way of addressing this research question, as this provides a tool for handling the large bias generated by a high number of iatrogenic onset of births within the group of women having undergone a previous CS. This approach shows that the dominant association between a CS and the duration of a subsequent pregnancy is the prolonging of it, which contrasts with previous research on this subject. This highlights the importance of choosing appropriate models for complex perinatal outcomes. While the new findings add insights about the relationship between CS and the gestational duration of subsequent pregnancies, the question if a causal relationship between the two exists is yet to be proved.

## Supporting information

S1 Table ICD-codes for variable definitions. ICD: International Classification of Diseases.
(DOCX)

S1 Fig Multivariable logistic regression analyses, first two pregnancies. Adjusted odds ratio for gestational age at birth within the specified interval, in the second pregnancy after cesarean section in the first pregnancy. Only mothers with spontaneous onset of birth and gestational duration of the second pregnancy estimated by ultrasound.
(TIF)

S2 Fig Multivariable logistic regression analyses, first three pregnancies. Adjusted odds ratio for preterm birth in the third pregnancy for the specified comparison between different sequences of previous delivery modes. Only mothers with spontaneous onset of birth and gestational duration of the third pregnancy estimated by ultrasound. *CS*: cesarean section, *Vag*: vaginal delivery.
(TIF)
