# Supplementary material for: Cesarean section and the gestational duration of subsequent pregnancies: A nationwide register-based cohort study
Source: PLoS One. 2025 Feb 5;20(2):e0317492. doi: 10.1371/journal.pone.0317492 (PMC11798495; doi:10.1371/journal.pone.0317492)
Supplement: S1 Table — ICD: International Classification of Diseases. (DOCX) [file pone.0317492.s001.docx]

| **Diagnosis** | **ICD-codes** |
| --- | --- |
| Diabetes | ICD-8: 250,00; 250,09; 761,10. ICD-9: 250A; 250B; 250C; 250D; 250E; 250F; 250X; 648A. ICD-10: E10.7; O24.0; O24.1; O24.3; O24.4; O24.9; O24.0C; O24.0D; O24.0E; O24.0F; O24.0X; O24.4A; O24.4B; O24.4X. |
| Hypertension | ICD-8: 401,99. ICD-9: 401; 401X. ICD-10: I10; I10.9. |
| Gestational hypertension | ICD-8: 637,01. ICD-9: 642; 642A; 642B; 642C; 642D; 642X. ICD-10: O13.9. |
| Preeclampsia | ICD-8: 637,03; 637,04; 637,10; 637,99; 762,10; 762,20; 762,30. ICD-9: 642E; 642F; 642G; 642H. ICD-10: O14; O14.0; O14.1; O14.2; O14.9; O14.1A; O14.1B; O14.1X. |
