# Supplementary figures and images for: Cesarean section and the gestational duration of subsequent pregnancies: A nationwide register-based cohort study
Source: PLoS One. 2025 Feb 5;20(2):e0317492. doi: 10.1371/journal.pone.0317492 (PMC11798495; doi:10.1371/journal.pone.0317492)

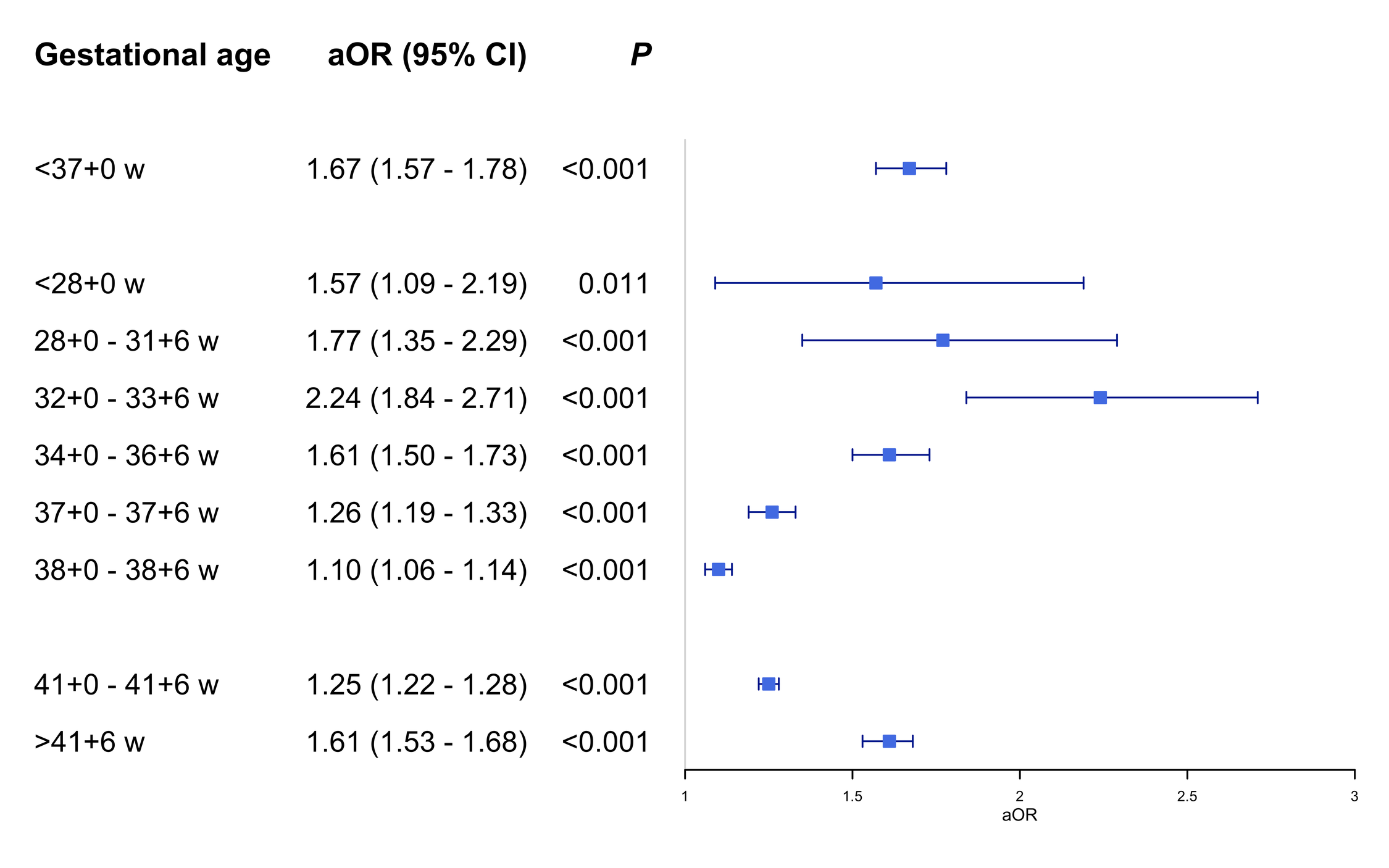

Supplement: S1 Fig — Adjusted odds ratio for gestational age at birth within the specified interval, in the second pregnancy after cesarean section in the first pregnancy. Only mothers with spontaneous onset of birth and gestational duration of the second pregnancy estimated by ultrasound. (TIF) [file pone.0317492.s002.tif]

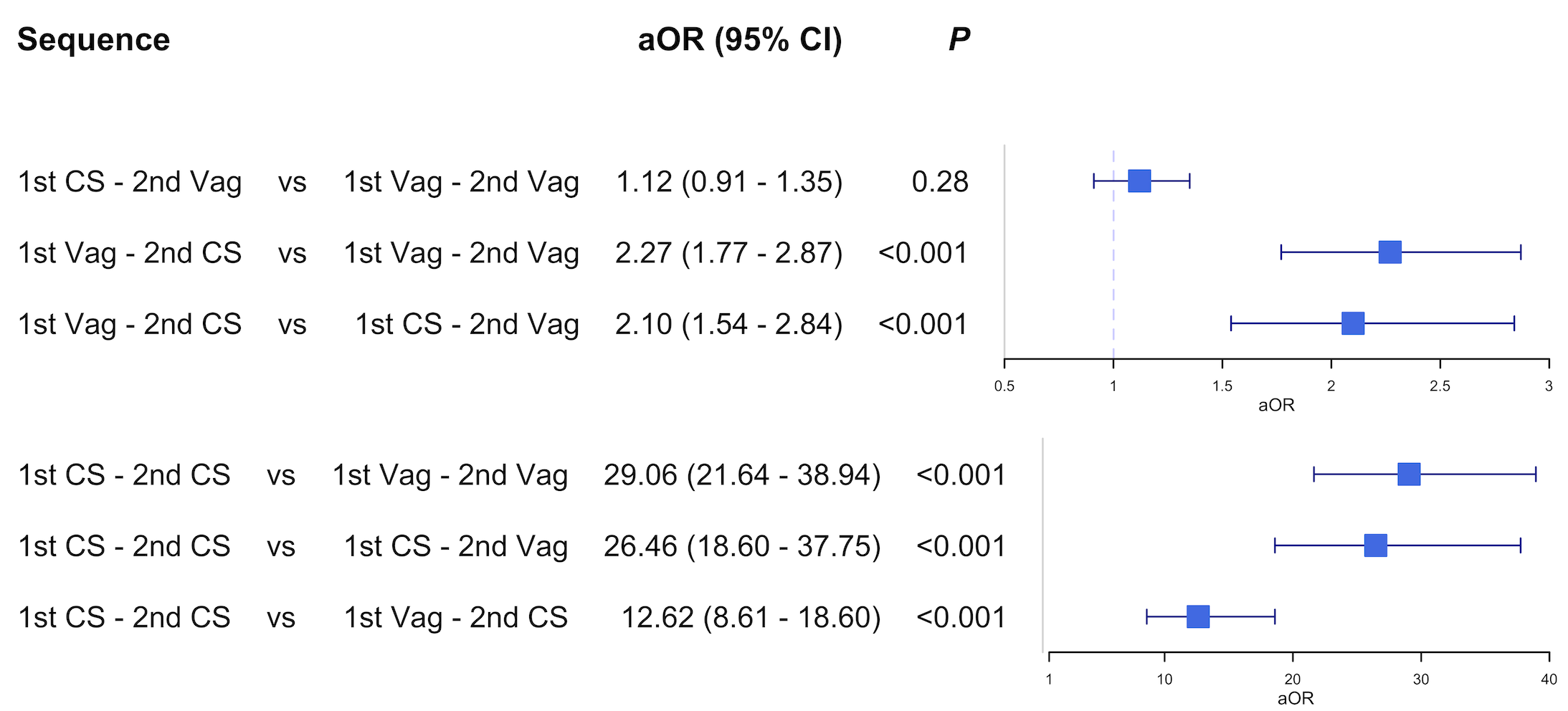

Supplement: S2 Fig — Adjusted odds ratio for preterm birth in the third pregnancy for the specified comparison between different sequences of previous delivery modes. Only mothers with spontaneous onset of birth and gestational duration of the third pregnancy estimated by ultrasound. CS: cesarean section, Vag: vaginal delivery. (TIF) [file pone.0317492.s003.tif]
